# Successful treatment of post‐operative peripheral bronchopleural fistulas using endobronchial Watanabe spigots

**DOI:** 10.1002/rcr2.504

**Published:** 2019-11-25

**Authors:** Shinichi Yamamoto, Masaya Sogabe, Hideki Negishi, Sayaka Mitsuda, Tomoki Shibano, Shunsuke Endo

**Affiliations:** ^1^ Department of General Thoracic Surgery Jichi Medical University Shimotsuke Japan

**Keywords:** Bronchial occlusion, digital drainage system, endobronchial Watanabe spigot, peripheral bronchopleural fistula

## Abstract

Post‐operative peripheral bronchopleural fistulas (BPF) are sometimes caused by post‐operative pneumonia and empyema. Conservative treatment options such as administration of antibiotics and chest tube drainage can have limited outcomes in certain cases. Bronchial occlusion is an effective treatment option if the target bronchi for BPF are identified. This case study describes a successful bronchial occlusion for peripheral BPF with endobronchial Watanabe spigots (EWSs) and a digital drainage system. This case involved a 70‐year‐old man who developed a post‐operative peripheral BPF after a left upper lobectomy. Bronchial occlusion with EWS was performed because the target bronchi responsible for BPF were clearly detected by a chest computerized tomography scan. The effectiveness of the occlusion was confirmed with the use of a digital drainage system immediately after the procedure was completed. The chest tube was removed one week following the bronchial occlusion procedure.

## Abstract 2

A 70‐year‐old man who developed a post‐operative peripheral bronchopleural fistulas (BPF) after a left upper lobectomy. Bronchial occlusion with endobronchial Watanabe spigots (EWS) was performed because the target bronchi responsible for BPF were clearly detected by a chest computerized tomography scan. The effectiveness of the occlusion was confirmed with the use of a digital drainage system immediately after the procedure was completed.

## Graphical abstract

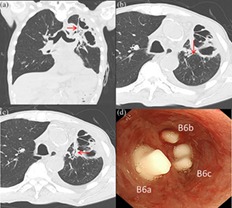

## Introduction

Post‐operative bronchopleural fistula (BPF) can occur as a serious complications of lung resection. This may result in increased morbidity and mortality for the patient. In the case of post‐operative peripheral BPF, bronchial occlusion can be an effective treatment if the target bronchi causing the BPF are identifiable. In this case report, we discuss a successful bronchial occlusion for post‐operative peripheral BPF with endobronchial Watanabe spigots (EWSs) and a digital drainage system.

## Case Report

**Figure 1:**
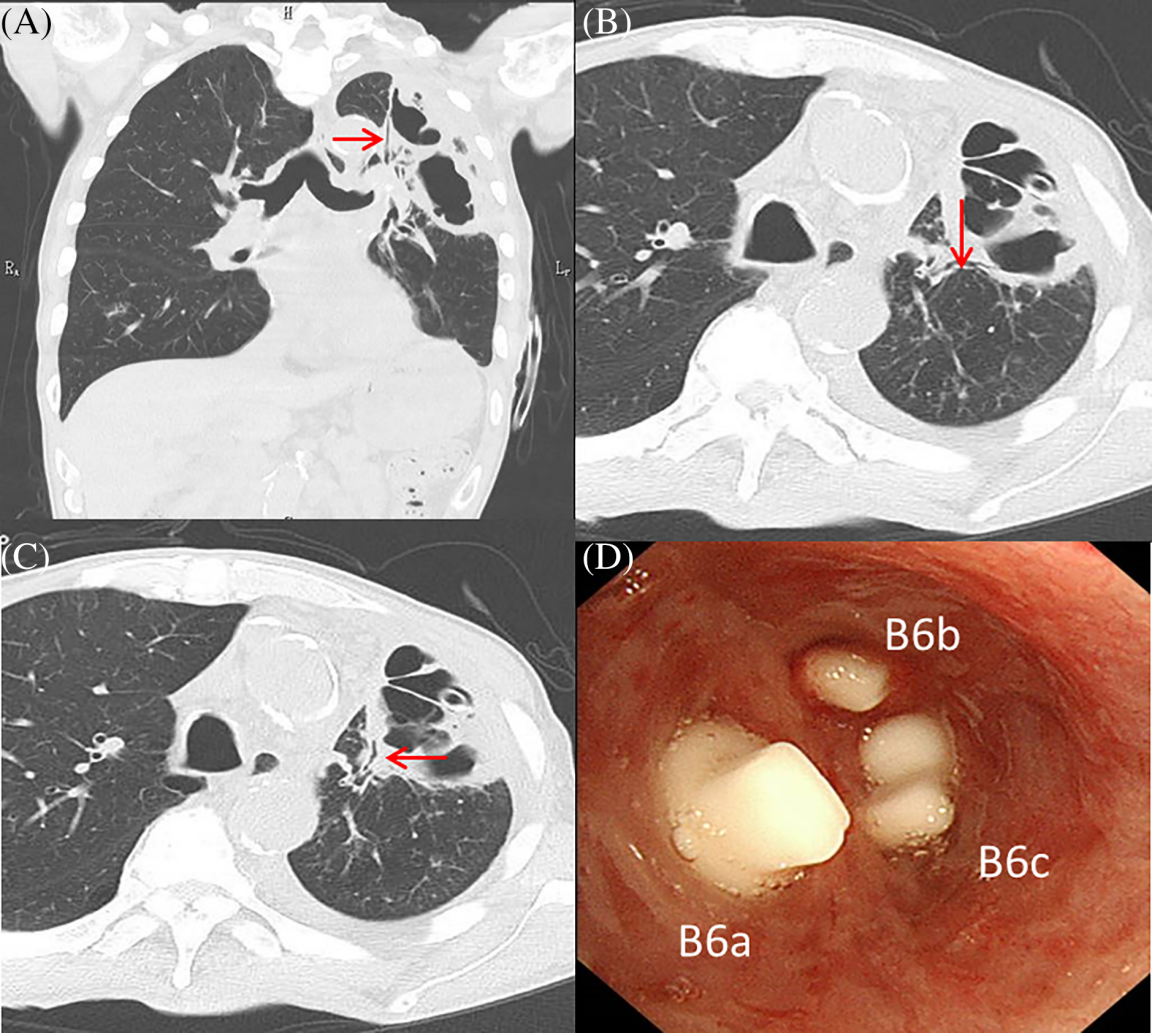
Chest computed tomography imaging shows the target bronchi for bronchopleural fistulas. (A) B6a; (B) B6b (C) B6c; (D) bronchoscopic findings after bronchial occlusion with endobronchial Watanabe spigots.

**Figure 2:**
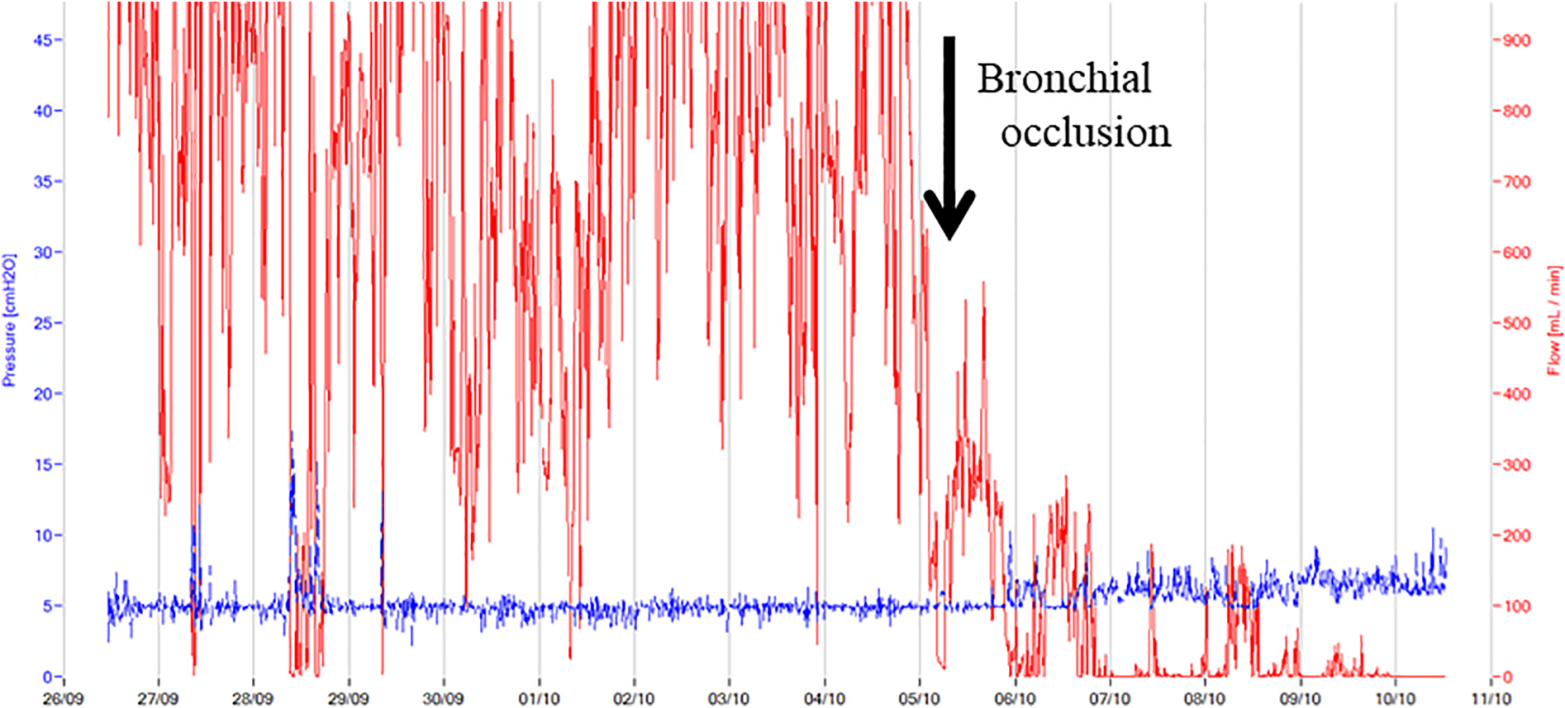
This chart shows the decrease in air leakage and the level of suction pressure. The red line shows the flow of air leakage (mL/min). The blue line shows the level of suction pressure (cmH_2_O).

A 70‐year‐old man with bladder cancer underwent an endoscopic bladder tumour resection. Prior to him undergoing this surgery, a full body scan was performed, in which a lung tumour in his left upper lobe was identified. He was diagnosed with metastatic lung cancer and was admitted to our institution for the resection. Video‐assisted thoracoscopic surgery was undertaken to perform the left upper lobectomy. The patient's chest tube was removed on post‐operative day 3 without any complications. On post‐operative day 6, the patient developed dyspnoea, and a chest X‐ray image showed his left lung had collapsed because of post‐operative pneumothorax. Chest tube drainage was commenced, and by post‐operative day 19, he developed left empyema due to pneumonia in the residual left lung. With antibiotic therapy, the patient's inflammatory markers improved. However, on post‐operative day 42, massive air leakage was identified. The chest computed tomography (CT) scan showed damage to the lung tissue in the superior segment of the left lower lobe and several bronchi leading to the intrathoracic cavity (Fig. 1A,B,C). We diagnosed post‐operative peripheral BPF. The left bronchi, B6a, B6b, and B6c, were identified using a three‐dimensional CT as the target bronchi for BPF. Bronchial occlusion with EWS (Novatech, Cedex, France) was performed immediately under local anaesthesia. A flexible bronchoscope, BF‐P‐290 (Olympus, Tokyo, Japan), and V‐shaped grasping forceps were used for the deployment of the EWSs. A digital drainage system (Thopaz, Medela, Baar, Switzerland) was used to confirm the effectiveness of the bronchial occlusion. EWSs were occluded in the left B6a, B6b, and B6c bronchi (Fig. 1D), and reduction in air leakage was achieved through the procedure (Fig. 2). The chest tube was removed one week after the bronchial occlusion when the air leakage had completely stopped. The patient did not want a repeat bronchoscopy, so he was examined subsequently in a six month clinical and CT imaging follow‐up and appeared to be in a good condition with no signs of BPF. We have no intention of removing deployed EWS unless the patient has complications such as pneumonia and lung abscess arising from EWS. Chest CT findings after six months showed no evidence of infection. If air leakage after removal reappears, we propose to use a combination of occlusion with re‐placement of EWS plus additional pleurodesis.

## Discussion

Post‐operative BPF can be a serious complication of lung resection and is associated with significant morbidity and mortality rates. It can be categorized as either central BPF or peripheral BPF. The common causes of peripheral BPF are necrotizing infections such as pulmonary tuberculosis, pneumonia, and empyema 1. In this particular case, peripheral BPF was caused by post‐operative pneumonia and empyema. Post‐surgery peripheral BPF should be monitored for and prompt treatment should be given if massive air leakage is suddenly detected in cases where patients have post‐operative pneumonia and empyema.

Commonly used surgical treatments, such as fenestration and thoracoplasty, can impair post‐operative quality of life, especially in patients with poor physical condition. It has been reported that bronchoscopic occlusion is effective for BPF and is less invasive than surgery 2. Identification of the target bronchi for BPF is important for the success of the bronchoscopic occlusion procedure 3.

In this case, we identified target bronchi through the use of a three‐dimensional CT scan and were able to achieve a good patient outcome by performing bronchial occlusion. Bronchial occlusion should be considered as the first treatment option when post‐operative BPF is detected and target bronchi are clearly identifiable.

EWS is widely used as an occlusion material, especially for treating peripheral BPF 2. Watanabe et al., who developed EWS, have reported the effectiveness of performing endobronchial occlusion in the bronchi to reduce air leakage 4. EWS is easy to deploy in the target bronchi by holding the EWS with grasping forceps via a flexible bronchoscope. EWS should be used for treating post‐operative peripheral BPF via bronchial occlusion in case where target bronchi are clearly identifiable.

A digital drainage system, such as the Thopaz, which was used in this case, provides objective real‐time data of the air leakage 5. We used the Thopaz to confirm the effectiveness of bronchial occlusion during the procedure. In this case, decreasing air leakage was visualized immediately after the bronchial occlusion. The amount of air leakage that we observed via the Thopaz helped to ensure the bronchial occlusion procedure was successful.

In conclusion, bronchial occlusion for peripheral BPF with EWS and a digital drainage system such as the Thopaz was an appropriate choice in this case. An important factor in the success of this procedure was identifying the target bronchi via CT scan and that the procedure was performed using appropriate instruments. Bronchial occlusion with EWS should be considered as the first treatment option when post‐operative BPF is detected and target bronchi are clearly identifiable.

### Disclosure Statement

Appropriate written informed consent was obtained for publication of this case report and accompanying images.

This study was conducted with the financial support of our department.
